# Gastric Outlet Obstruction and Iron Deficiency Anemia Secondary to Gastric Bezoar: A Case Report

**DOI:** 10.7759/cureus.35876

**Published:** 2023-03-07

**Authors:** Emily A Schofield, Valerie Vazquez, Jennifer Demuro, Daniel Lynch, Darwin Ang

**Affiliations:** 1 Medical School, University of Central Florida College of Medicine, Orlando, USA; 2 General Surgery, University of Central Florida College of Medicine, Ocala, USA; 3 General Surgery, HCA Healthcare, Ocala, USA; 4 Trauma, HCA Healthcare, Ocala, USA; 5 Surgery, University of Central Florida College of Medicine, Orlando, USA

**Keywords:** : gastric outlet obstruction, trichotillomania, iron deficiency anemia (ida), trichophagia, gastric bezoar

## Abstract

Trichobezoars are an accumulation of undigested hair in the gastrointestinal system. They are a rare finding and are more likely in young females. Diagnosis is largely dependent on history taking and imaging, and treatment involves the removal and psychiatric evaluation.

We describe the case of a 21-year-old female with a history of gastroesophageal reflux disease (GERD) who presented with abdominal pain. Imaging showed a distended stomach with a suspected swallowed foreign substance. The patient subsequently underwent midline laparotomy, gastrotomy, and bezoar extraction. Postoperatively the patient was found to have trichotillomania, trichophagia, anxiety, depression, and symptomatic anemia.

Initial management of gastric bezoars includes proper removal, but the additional follow-up needs to include psychiatric evaluation and treatment to prevent reoccurrence. It is also imperative to assess and treat underlying nutritional deficiencies.

## Introduction

Bezoars are an accumulation of indigestible substances in the gastrointestinal tract that are categorized based on their composition. Phytobezoars are made up of vegetable and fruit fibers, trichobezoars are made up of hair, and pharmacobezoars are made up of undigested medications. Trichobezoars are typically associated with young females who are affected by trichotillomania, a disorder characterized by the compulsive urge to pull out their own hair, or trichophagia, a disorder characterized by repeated ingestion of hair [1]. It is estimated to have an incidence of 0.068% [2].

Patients can present with a variety of symptoms, including abdominal pain, nausea, vomiting, fullness, difficulty swallowing, and weight loss [3]. Complications from bezoars include perforation, peritonitis, gastrointestinal obstruction, intussusception, and pancreatitis, but it largely depends on bezoar location [4,5]. The most common location is the stomach [3]. Diagnosis is mostly based on patient history, but it can also be based on imaging, including ultrasound, computerized tomography (CT), and abdominal X-rays, with CT imaging being the superior imaging modality [6].

Treatment options largely depend on size, location, and type of bezoar. For gastric trichobezoars, treatments include endoscopic removal, laparoscopic removal, and gastrotomy [7]. It is important to address the underlying cause of the bezoar to prevent recurrence as many patients with trichobezoars have psychiatric conditions [8]. Some of these include trichotillomania, obsessive-compulsive disorder, and depression [8]. In this report, we present a case of a trichobezoar causing gastric outlet obstruction leading to a diagnosis of trichotillomania and anemia.

## Case presentation

A 21-year-old female with a past medical history of gastroesophageal reflux disease (GERD) presented to the emergency department complaining of left-sided abdominal pain beginning a week and a half prior. The patient described the pain as sharp and located under her left lower rib cage that radiated to her left shoulder. She reported that the pain was worse with deep inhalation and when laying on her left side. She also reported decreased appetite. She denied any fever, chills, nausea, vomiting, shortness of breath, cough, syncope, headache, constipation, and diarrhea.

The patient’s past medical history was significant for GERD and had no other past medical history. The only current medication was omeprazole. The patient had no past surgical history. The patient was single, employed, and lived with her mother, stepfather, and sister. She denied ever smoking, alcohol use, or illicit drug use.

The patient’s vitals at initial presentation to the emergency department was a temperature of 36.4 °C, respiratory rate of 18 breaths per minute, blood pressure of 134/73 mm Hg, pulse rate of 89 beats per minute (bpm), and pulse oximetry of 97% on room air. 

A physical exam showed the patient awake and alert and in no acute distress. The head was atraumatic, the airway was patent, and mucous membranes were moist. The respiratory exam showed lungs clear to auscultation bilaterally. A cardiac exam revealed normal S1 and S2 with no rubs, gallops, or murmurs. The vascular exam showed 2+ pulses in the upper and lower extremities equal bilaterally. The abdominal exam was tender to palpation in the epigastric region and nondistended and with no guarding or rebound. The musculoskeletal exam showed a normal range of motion and nontender joints. The neurologic exam showed normal speech and no motor or sensory deficits. The skin was warm, dry, and intact. The psychiatric exam showed normal affect and mood. Laboratory tests are shown in Table 1. 

**Table 1:** Laboratory test results. CMP, complete metabolic panel

Laboratory test	Result	Reference values
White blood cells	7.9 × 10^3^ microL^-1^	4.5 × 10^3^ to 11 × 10^3^ microL^-1^
Hemoglobin	9 g/dL	12-16 g/dL
Hematocrit	28.5%	36%-46%
CMP panel	Within normal limits	
Pregnancy test	Negative	Negative
Urine ketones	40 mg/dL	Negative
Glucose	70 mg/dL	70-110 mg/dL
Hepatic function testing	Within normal limits	
Lipase	71 L^-1^	23-300 L^-1^

**Figure 1:**
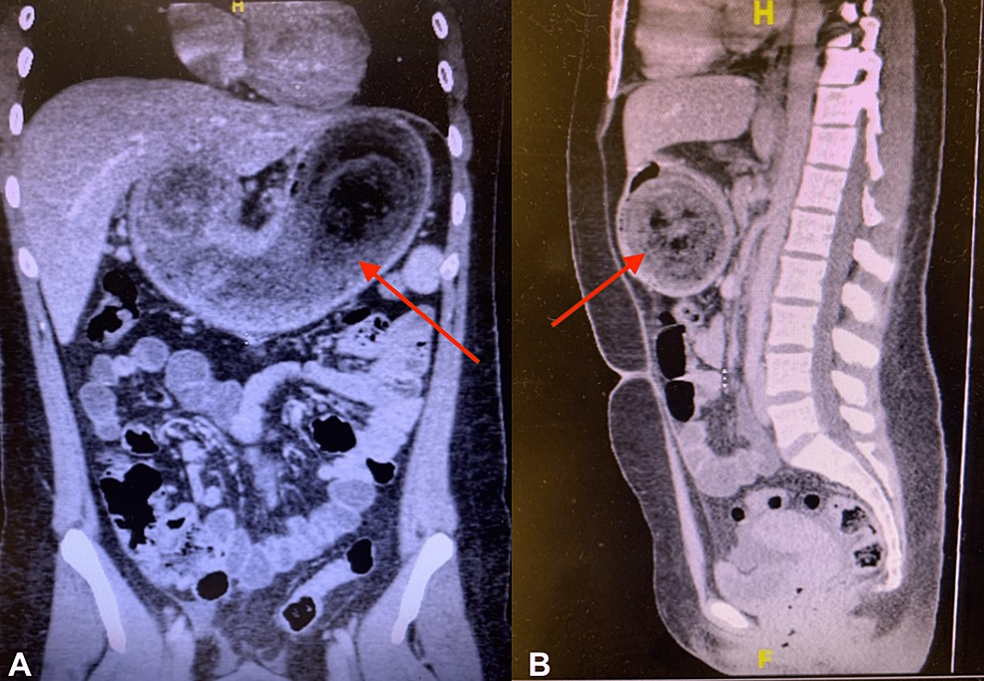
(A) Coronal CT scan showing a large mass in the gastric lumen depicted by the arrow; (B) sagittal CT image showing a large mass in the gastric lumen depicted by the arrow. CT, computed tomography

A CT of the chest showed no signs of pulmonary embolism or other acute findings. A CT of the abdomen and pelvis with contrast showed an abnormal gastric lumen distension with a suspected large, ingested debris questionable for an obstructing bezoar causing possible gastric outlet obstruction (Figure 1). The CT also showed mild pelvic free fluid without signs of pneumoperitoneum. 

Given the CT report and imaging depicting a large bezoar, the patient was asked further about the consumption of hair. She then revealed that she occasionally chewed on hair but denied swallowing it. General Surgery and Gastroenterology were consulted. Because of the size of the bezoar, it was decided that endoscopy would be unsuccessful, and the patient consented to a midline laparotomy, gastrotomy, and bezoar extraction.

**Figure 2:**
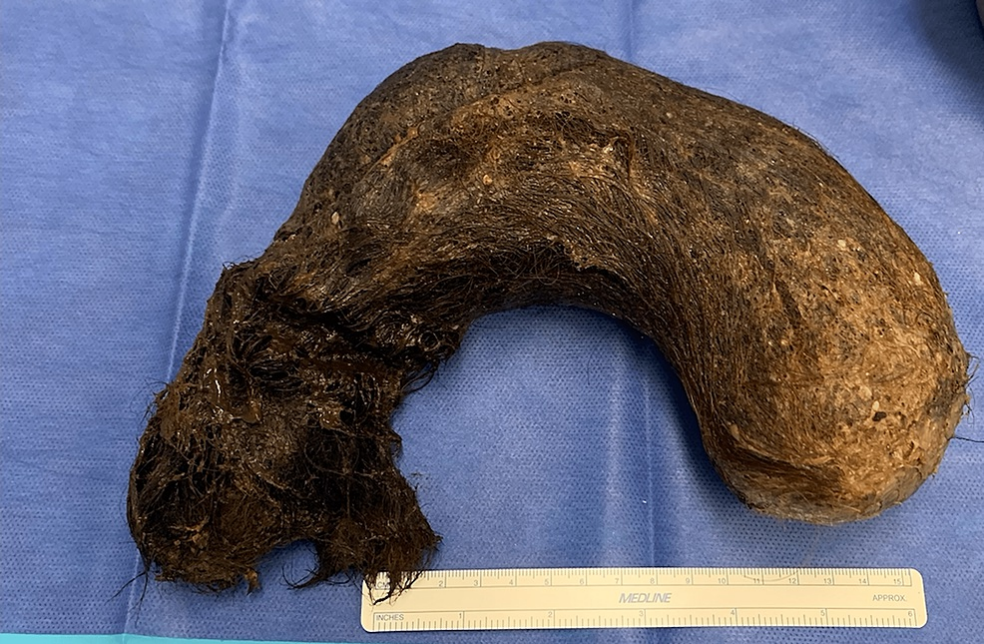
Gastric bezoar with dimensions 23.5 cm × 20 cm × 9.1 cm extracted from the patient's stomach.

The patient was told to take nothing by mouth (NPO), started intravenous (IV) fluids, and a nasogastric tube (NG tube) was placed. The following morning, the patient was taken to the operating room, underwent general endotracheal anesthesia, and a midline laparotomy, gastrotomy, and bezoar extraction were completed. Preoperative antibiotics were given, and a Foley catheter was placed. A 3 cm gastrotomy was performed using electrocautery, and the bezoar was immediately identified. Pieces of the bezoar were initially extracted with ring forceps, and then with gentle traction, the entire bezoar was removed in whole (Figure 2). The bezoar was found to have dimensions of 23.5 cm × 20 cm × 9.1 cm and filled the gastric cavity. The stomach mucosa was evaluated and was determined to be nonischemic and healthy. After ample irrigation, the stomach was closed and reinforced with Lembert (Ethicon, Raritan, NJ, USA) sutures. A tongue of omentum was brought up over the gastrotomy closure for a buttressing reinforcement and secured with three 2.0 Silk (Ethicon) sutures. The fascia was closed with two number 1 running Polydioxanone (PDS II, Ethicon) sutures, and the skin was closed with running 4.0 Monocryl (Ethicon) subcuticular sutures. The patient tolerated the procedure well and was taken to the postanesthesia care unit (PACU). 

The following day, the psychiatric service was consulted and reported that the patient had been pulling her hair out since 2017, had depressive symptoms since 2019, and had increased anxiety. The patient was then started on Prozac (Major Pharmaceuticals, Livonia, MI, USA) 20 mg once daily for trichotillomania, depression, and anxiety and asked to follow up outpatient.

The patient was afebrile with stable vital signs, and her incision was clean, dry, and intact, with some diffuse abdominal pain on palpation. On postoperative day 2, the patient was afebrile but became tachycardic to 149 bpm and became diaphoretic, which was determined to be secondary to anemia. On postoperative day 3, iron, folic acid, vitamin B12, and hemoglobin studies were ordered and were remarkable for iron less than 10 mcg/dL, hemoglobin of 7.1 g/dL, vitamin B12 of 260 pg/mL, and folic acid within normal limits. On postoperative day 4, she was treated with Ferrlecit ×1 dose (Hikma Pharmaceuticals, London, UK), oral iron replacement, and vitamin C 2 g twice a day. Hematology was consulted due to her anemia, and the patient was diagnosed with iron deficiency anemia, most likely secondary to trichotillomania impairing the absorption of iron and vitamin B12. The patient was then started on IV Feraheme (Hikma Pharmaceuticals) 125 mg transfusion daily for five days and 1,000 mcg of vitamin B12 supplementation per day as an outpatient. The patient improved clinically and was discharged after five days of iron transfusion.

## Discussion

We report the case of a 21-year-old female presenting with a week and a half of right upper quadrant abdominal pain, which was found to be a giant gastric bezoar with gastric outlet obstruction. It was determined by imaging and further questioning that the patient was affected by trichotillomania, trichophagia, depression, and anxiety. Postoperatively, the patient became diaphoretic and tachycardic, which was determined to be secondary to anemia. She was then treated for her anemia with iron transfusions with vitamin B12 supplementation and was discharged five days later.

Trichotillomania is a common condition with a prevalence of 0.6% to 3.6% in the general population [9]. It is more commonly found in female patients and usually starts in adolescence, with males having an earlier onset [10]. In adult cases, females account for about 90% of the cases, while in pediatric cases, females account for 75% [10]. Over 20% of trichotillomanic patients also have trichophagia, and some of these patients will require surgical intervention [11]. Diagnosis of trichotillomania is based on the Diagnostic and Statistical Manual of Mental Disorders, Fifth Edition (DSM 5) definition, which includes five criteria: hair loss, resulting from recurrent pulling out of one's hair; multiple efforts to stop or reduce episodes of pulling out one's hair; clinically significant distress or impairment in social, occupational, or other important areas of functioning from hair pulling; no medical conditions associated with the hair pulling or hair loss; and no symptoms of mental disorders associated with the hair pulling [12]. Treatment typically includes selective serotonin reuptake inhibitors (SSRIs), clomipramine, and cognitive behavioral therapy, although more research is needed to determine the best course of treatment [13]. SSRIs and clomipramine are considered first line, but SSRIs have not shown great efficacy over other treatments, but are commonly chosen in patients with concurrent mood disorders [13]. Trichophagia was the cause of this patient’s trichobezoar and was addressed after surgery by the psychiatric team with an SSRI (Prozac) due to concurrent depression and anxiety and follow-up therapy.

This case highlights important factors when treating patients with trichobezoars. First is the importance of obtaining a complete history. This patient did not initially reveal her tendencies to pull out and eat hair until specifically asked. It is also important to find the root cause of this behavior to prevent reoccurrence. It was discovered with further discussion with this patient that she was affected by depression and anxiety.

In the case of chronic trichophagia and bezoar removal, nutritional deficiencies should be assessed. Hair is indigestible and has an enzyme-resistant surface, allowing it to accumulate in the stomach and causing gastric obstruction [8,14]. With a large indigestible mass in the stomach for years, limited food digestion and absorption occur. In this case, the patient became symptomatically anemic. Once she started tolerating a normal diet, she had very low iron stores, which needed to be replaced. She also had moderate ketones in her urine, further suggesting a state of ketosis and malnutrition. During long-term fasting or long periods of malnutrition due to maldigestion and malabsorption, the body adapts by making and using ketones, limiting the amount of glucose consumed [15].

## Conclusions

Although uncommon, gastric trichobezoars should be included in a differential diagnosis in young female patients who present with abdominal pain and anemia. We present a rare condition in which surgery was required to remove a giant bezoar secondary to trichophagia. Misdiagnosis could lead to major health complications that could require emergent surgery and chronic malnutrition. This condition requires good history taking and a multidisciplinary approach for diagnosis and treatment.
